# Potential Beneficial Effects of Wine Flavonoids on Allergic Diseases

**DOI:** 10.3390/diseases7010008

**Published:** 2019-01-15

**Authors:** Toshio Tanaka, Atsuhiko Iuchi, Hiroshi Harada, Shoji Hashimoto

**Affiliations:** 1 Department of Cardiology, Osaka Prefectural Hospital Organization Osaka Habikino Hospital, Osaka 583-8588, Japan; mail123@ra.opho.jp (A.I.); harada-hi@ra.opho.jp (H.H.); 2 Department of Clinical Laboratory, Osaka Prefectural Hospital Organization Osaka Habikino Hospital, Osaka 583-8588, Japan; hashisyo@ra.opho.jp

**Keywords:** allergy, antioxidant, wine flavonoids

## Abstract

Wine, a widely consumed beverage, comprises several biophenols that promote health. Flavonoids, majorly present in red wine, have been shown to have antioxidant, anti-inflammatory, anticancer, and immunomodulatory activities. Regular consumption of red wine (100 mL/day) is estimated to provide an average of 88 mg of flavonoids, whereas recent epidemiological studies indicate that wine is one of the major sources of flavonoid intake amongst wine lovers in European countries (providing an average intake of 291–374 mg/day of flavonoids). In addition to being antioxidants, in vitro studies suggest that flavonoids also have anti-allergic activities that inhibit IgE synthesis, activation of mast cells and basophils or other inflammatory cells, and production of inflammatory mediators, including cytokines. Furthermore, they affect the differentiation of naïve CD4+ T cells into effector T cell subsets. Moreover, several studies have reported the benefits of flavonoids in allergic models such as atopic dermatitis, asthma, anaphylaxis, and food allergy; however, evidence in humans is limited to allergic rhinitis and respiratory allergy. Although further evaluation is required, it is expected that an appropriate intake of flavonoids may be beneficial in preventing, and eventually managing, allergic diseases.

## 1. Introduction

The prevalence and incidence of allergic diseases, such as allergic rhinitis, asthma, atopic dermatitis, and food allergy, have increased worldwide during the past two to three decades [1,2]. The environmental and genetic interaction leads to sensitivity in individuals towards environmental allergens, then causes allergic diseases [3,4,5]. The “diet hypothesis” proposes that changes in dietary habit may play a significant role in the increase, since foods and beverages contain allergy-promoting and anti-allergic nutrients [6,7,8]. Minerals such as selenium, copper, zinc, and magnesium, vitamins A, C, D, and E, probiotics, and omega-3 polyunsaturated fatty acids (PUFAs) possess anti-allergic functions, whereas omega-6 PUFAs are precursors for leukotriene C4, which promotes allergic inflammation [6].

Flavonoids, polyphenolic plant secondary metabolites, have antioxidant, anti-inflammatory, and anti-allergic activities as well as immunomodulating effects [9,10]. Red wine, a major source of flavonoids for wine lovers, is known to reduce cardiovascular events when consumed in moderation [11]. Although the benefits of red wine in allergic diseases have not been elucidated in detail, based on recent findings, the present article emphasizes that an appropriate intake of flavonoids may be beneficial in preventing, and eventually managing, allergic diseases.

## 2. Flavonoids, the Major Ingredient in Red Wine for Promoting Health

**Figure 1:**
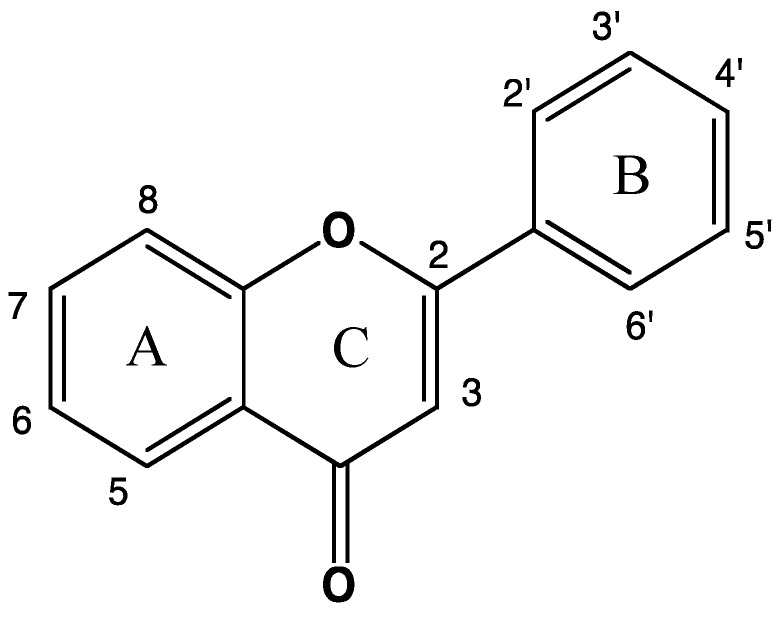
Structure of basic flavonoid skeletons.

Flavonoids are found in fruits, vegetables and tea, thus forming common ingredients of the daily diet [12,13,14]. Flavonoids, which share a common structure comprising two aromatic rings (A and B) bound together by three carbon atoms forming an oxygenated heterocycle (ring C) (Figure 1), are generally classified into six subclasses: flavones (luteolin, apigenin, and baicalein), flavonols (fisetin, kaempferol, quercetin, myricetin, and isohamnetin), flavanones (hesperetin, naringenin, and eriodictyol), isoflavones (daidzein and genistein), anthocyanidins (cyanidin, delphinidin, malvidin, pelargonidin, petunidin, and peonidin) and flavanols (catechins and proanthocyanidins).

Wine is a dietary source of phenolic compounds, namely flavonoids and non-flavonoids, which include phenolic acids, phenols, and stilbenes [11]. Recent developments regarding the flavonoid content of foods and beverages in the databases of the US Department of Agriculture (USDA) [15], the European BioActive Substances in Food Informative System (EuroFIR-BASIS) [16], and the Phenol-Explorer [17,18] have led to epidemiological studies precisely aiming to clarify the association between flavonoid intake and the prevalence and incidence of chronic diseases and cancers. According to the Phenol-Explorer database, the average intake of total flavonoids in France is 506 mg/day (with 51 mg/day of flavonols and 33 mg/day of flavones) [19], in the Mediterranean countries is 370.2 mg/day (with 24.8 mg/day of flavonols and 5.6 mg/day of flavones), and in the non-Mediterranean countries is 373.7 mg/day (with 29.5 mg/day of flavonols and 4.1 mg/day of flavones) [20]. This shows that the total daily consumption of flavonoids is higher in France than that in the other European countries. The same database indicates that 100 mL of red wine on average includes 88 mg of flavonoids, comprising anthocyanins (28 mg), dihydroflavonols (5.4 mg), flavanols (47 mg), flavanones (0.9 mg), and flavonols (6.9 mg) (Table 1), which may vary depending on the source and ageing, while white wine includes considerably less flavonoids (3.5 mg/100 mL). The USDA database for the flavonoid content of selected foods, release 3.3 (March 2018), reports that red wine includes 34.5–171.9 mg of flavonoids per 100 g, depending on the source [15].

Several epidemiological studies have reported a positive association between red wine intake and health. Individuals who consume moderate amounts of wine experience 20–30% reductions in all-cause mortality, particularly cardiovascular mortality [21], an effect known to be associated with the flavonoid composition of red wine [22]. The “French paradox” refers to the reduced cardiovascular mortality, due to higher intakes of red wine in France, when compared with other countries that consume similar amounts of saturated fats [23]. This preventive effect is considered to be based upon the strong antioxidant capacity of red wine flavonoids [11], since they react with the reactive compound of the radicals, and stabilize the reactive oxygen species [24,25].

## 3. Anti-Inflammatory and Anti-Allergic Activities of Flavonoids Observed by In Vitro Experiments

The research provides evidence that oxidative stress is crucial in the airway and skin inflammation observed in asthma and atopic dermatitis patients, respectively [26,27]. The strong antioxidant capacity of flavonoids suppresses this allergic inflammation. Additionally, flavonoids are known to exert various ameliorative effects on allergic diseases [28,29].

Allergy is an IgE-mediated disease, pathologically comprising the sensitization and the effector phases. Flavonoids possess anti-allergic properties affecting both phases. Fewtress and Gomperts first identified the inhibition by flavones of transport ATPase in histamine release from rat mast cells [30]. Subsequently, flavonoids have been shown to inhibit the release of chemical mediators, such as histamine, hexosaminidase, and cyteinyl leukotrienes, by rat mast cells or human basophils [31,32,33]. In addition to the release of chemical mediators, mast cells and basophils can produce several cytokines associated with the late-phase allergic reaction. Meanwhile, flavonoids such as luteolin, quercetin, and baicalein were found to inhibit the synthesis of granulocyte macrophage-colony stimulating factor, tumor necrosis factor-α, and interleukin (IL)-6 production by the cultured mast cells in response to the cross-linkage of a high-affinity IgE receptor (FcεRI) [34,35]. IL-4 plays a major role in the sensitization phase since it stimulates the differentiation of B cells into IgE-producing cells and promotes the differentiation of naïve T cells into Th2 cells. Then, we examined the inhibitory effects of 45 kinds of flavonols and their related compounds on IL-4 synthesis, by analyzing the purified human peripheral blood basophils in response to cross-linkage of FcεRI [36,37,38]. Luteolin, apigenin, and fisetin showed the strongest inhibitory activity, with the half-maximal inhibitory concentration (IC_50_) value of these flavonoids for IL-4 synthesis ranging from 2.7–5.8 μM. Quercetin and kaempferol, meanwhile, had a moderate inhibitory effect on the IL-4 synthesis, with an IC_50_ value of 15.7–18.8 μM. Moreover, kaempferol was demonstrated to suppress the activation of IL-4 receptor-mediated signal transducers and activators of transcription, (STAT)6, by targeting Janus kinase (JAK)3 in the hematopoietic cell line [39]. Furthermore, epigallocatechin gallate, epicatechin gallate, gallocatechin gallate, anthocyanindin, delphinidin, and tricetinidin possess a pyrogallol function that suppresses the expression of FcεRI on human mast cells [40].

The aryl hydrocarbon receptor (AhR) is a receptor that leads to the toxic and biological actions of several aromatic environmental pollutants, such as dioxin [41]. In vitro bioassay of the dioxin (2,3,7,8-tetrachlorodibenzo-*p*-dioxin (TCDD)) revealed that flavonoids including apigenin, luteolin, baicalein, quercetin, kaempferol, and myricetin had significant inhibitory effects on the AhR activation, with an EC_70_ value (equal to 70% of the maximal response to TCDD) of 1.9–5.1 μM [42]. It has been demonstrated that the activation of AhR interferes with the differentiation of naïve CD4+ T cells into effector T cell subsets [43,44,45,46].

Nuclear factor-kappa B (NF-κB) is an important transcriptional factor that contributes pathologically to the development of various inflammatory diseases, including asthma, by inducing inflammatory responses, cell adhesion, and the anti-apoptosis process [47]. Flavonoids are also shown to suppress the NF-κB activation [48].

Autophagy is a cellular pathway that maintains cell homeostasis by eliminating the damaged cellular components, and its dysregulation may be associated with the development of various diseases [49]. The role of autophagy is also demonstrated in severe asthma, and flavonoids could potentially constitute the important adjuvants of conventional therapies for treating autophagy-related diseases [50].

## 4. Effects of Flavonoids on Allergic Diseases

As mentioned above, based on several anti-allergic activities of flavonoids, it is anticipated that an appropriate intake of flavonoids might prove beneficial in treating allergic diseases [51]. Indeed, the administration of flavonoids has revealed preventive or therapeutic effects in several allergy models.

We examined the preventive effect of astragalin (kaempferol 3′glucoside) on the onset or development of dermatitis by using NC/Nga mice, a model of atopic dermatitis [52]. The mice, which were administered a control diet, exhibited symptoms of dermatitis, scratching behavior, and serum IgE elevation along with aging, whereas the oral administration of astragalin (1.5 mg/kg) markedly prevented these symptoms [53]. Moreover, administrating an extract from the petals of *Impatiens balsamina* L., containing kaempferol 3-rutinoside and 2-hydroxy-1,4-naphthoquinone [54], prevented the development of dermatitis, while apigenin [55] and baicalein [56] therapeutically improved the severity of dermatitis in NC/Nga mice.

It was further demonstrated that in an ovalbumin (OVA)-sensitized asthmatic mouse model, the oral intake of luteolin (0.1 mg/kg) inhibited the bronchial hyper-reactivity and bronchoconstriction [57]. Moreover, it was reported that a polymethoxyflavonoid nobiletin, when administered at a dose of 1.5 or 5 mg/kg intraperitoneally to the OVA-sensitized rats, could reduce the number of eosinophils and the expression of eotaxin [58]. Subsequent investigations reported that numerous flavonoids such as quercetin, isoquercitrin, rutin, 3-*O*-methylquercetin 5,7,3′,4′-*O*-tetraacetate, narirutin, apigenin, luteolin, sulfuretin, hesperdin, fisetin, kaempferol, acacetin, silibinin, naringin, limonene, chrysin, genistein, skullcapflavone II, and anthocyanins indicated improvement in the asthmatic models [59]. Moreover, quercetin effectively quelled the anaphylactic reaction in the peanut-sensitized rats [60].

Several epidemiological studies have assessed the association of flavonoid intake with allergic diseases. A cohort study of the association between flavonoid intake and chronic diseases on 10,054 adults in Finland reported that the asthma incidence was lower with higher quercetin and hesperetin intakes [61]. A population-based case–control study performed in South London, UK, wherein 607 cases and 864 controls were enrolled, indicated that apple consumption was negatively associated with asthma, whereas red wine intake was negatively associated with asthma severity [62]. The authors speculated that the associations between apple and red wine consumption and asthma might indicate a protective effect of flavonoids. However, there is a need to be careful as alcoholic drinks, particularly wines, have been shown to be associated with the triggering of asthma in respondents [63]. A subsequent study by the same research group, however, did not find any significant association of the dietary intake of catechins, flavonols, and flavones with the asthma prevalence and severity in a case–control study of 1471 adults in London [64]. The GA^2^LEN (Global Allergy and Asthma European Network) study investigated the role of six major subclasses of flavonoids on ventilator function, with 2599 adults (aged 15 to 75 years) from nine European countries were enrolled [65]. The general consumption of 250 food types was estimated by the GA^2^LEN food frequency questionnaire, and the intake of six major flavonoid subclasses; flavanones (eriodictyol, hesperetin, and naringenein), anthocyanins (cyaniding, delphinidin, malvidin, pelargonidin, petunidin, and peonidin), flavanols (catechins and epicatechins), flavonols (quercetin, kaempferol, myricetin, and isohamnetin), flavones (luteolin and apigenin) and polymers (proanthocyanidins, theaflavins, and thearubigins), and proanthocyanidins was calculated using the USDA database. The average of the total flavonoid intake was 291.2 mg/day and it varied among people from the nine countries (from 231.7 mg/day in Germany to 817.3 mg/day in Poland), whereas the intake of proanthocyanidins was 154.6 mg/day. Among the total food and beverage consumption, wine and beer together contribute to about 21% and 14.9% of the total flavonoid and proanthocyanidin intake, respectively. A lower prevalence of forced vital capacity (FVC) below the lower limit of normal and a higher ratio between forced exhaled volume in 1 second (FEV_1_) and FVC (FEV_1_/FVC) was observed in those with higher total flavonoid and proanthocyanidin intakes.

Nevertheless, flavonoid intervention in humans is limited. Previous clinical research using several flavonoid extracts indicates that flavonoids have therapeutic effects on allergic rhinitis [66,67,68,69,70]. These extracts were *Perilla frutescens* (rosmarinic acid as a major flavonoid), apple polyphenols (procyanidins or apple condensed tannin, catechin, epicatechin, phlorizin, and chlorogenic acid), hop water extract (quercetin and kaempferol glycosides), and tomato extract (naringenin chalcone). A summary of these flavonoid intervention studies in allergic rhinitis is shown in Table 2. Enzymatically-modified isoquercitrin (EMIQ) is a quercetin glycoside comprising isoquercitrin and its maltooligosaccharides, which markedly enhances the bioavailability. We performed clinical research to examine the efficacy of EMIQ on patients with Japanese cedar pollinosis in 2007 and 2008 [71,72]. In a double-blind, placebo-controlled design, the patients were randomly assigned to the EMIQ group or the placebo group. The 2007 study commenced after the pollen dispersion, and thus we examined the therapeutic effect of EMIQ, whereas the 2008 study commenced 3 weeks before the first day of pollen dispersion, to evaluate the preventive effect of EMIQ on the symptoms of pollinosis. The daily intake for these studies was 100 mg EMIQ for 8 weeks. The total symptom (nasal and ocular symptoms) scores for the EMIQ groups in the 2007 and 2008 trials were optimally lowered by 48% and 33%, respectively, compared with the scores for the placebo groups, indicating a substantial ameliorative effect of EMIQ. A randomized clinical trial of silymarin demonstrated its ameliorative effect on the symptoms of allergic rhinitis [73]. Moreover, a randomized, double-blind, placebo-controlled study of pycnogenol, a proprietary mixture of water-soluble bioflavonoids extracted from the French maritime pine, which contains proanthocyanidines, revealed its ameliorative effect on seasonal allergic rhinitis [74].

Pycnogenol was also demonstrated to be effective in treating asthma. The first study was performed to evaluate the effect of pycnogenol on asthma in a randomized, double-blinded, placebo-controlled, crossover design, in which 26 asthmatic patients were enrolled [75]. These patients were randomly assigned to receive either 1 mg/lb/day (maximum 200 mg/day) pycnogenol or a placebo for 4 weeks and were then crossed over to the other regimen for the next 4 weeks. Twenty-two patients who completed the study responded positively to pycnogenol. Subsequently, in a randomized, placebo-controlled, double-blind study involving 60 asthmatic patients, aged 6–18 years, compared with the placebo group, the pycnogenol group revealed significantly greater improvement in the lung function and asthmatic symptoms, which resulted in the reduced or discontinued use of rescue inhalers [76]. Another study, which evaluated the effect of pycnogenol on the allergic asthma management of patients for 6 months, also revealed a favorable result [77]. In this study, pycnogenol at 100 mg/day proved to be effective in controlling the symptoms of allergic asthma and reduced the need for medication.

## 5. Future Perspectives of Red Wine Flavonoids for Allergic Diseases

A direct interventional study evaluating the beneficial effects of red wine flavonoids on allergic diseases has not been performed to date. However, as described elsewhere, one epidemiological study reported that red wine intake was negatively associated with asthma severity and suggested that flavonoids may produce a protective effect on asthma. Red wine is a major source contributing to the daily flavonoid intake for wine lovers, thus possibly ameliorating the allergic symptoms. However, careful attention is required in clinical trials, as wine is a triggering factor for worsening symptoms in certain asthmatic patients and heavy wine consumption is accompanied by alcohol intake that is not good for health and behavior [78].

Table 3 summarizes the anti-allergic effects of flavonoids. Flavonoids possess antioxidant, anti-inflammatory, anti-allergic, and immunomodulating effects. Several studies have reported the benefits of flavonoids in allergic models, however, the evidence in the epidemiological studies and clinical studies is presently limited. Future studies are needed, to focus on whether an appropriate intake of flavonoids can constitute a dietary contribution in the prevention and amelioration of allergic diseases.

## Figures and Tables

**Table 1:** Contents of flavonoid family and major flavonoids in red wine.

Data Source	Phenol-Explorer (mg/100 mL) Mean (min~max) [18]	USDA (mg/100 g) Mean (min~max) [15]			
	Red Wine	Red Wine	Red Wine, Cabernet Franc	Red Wine, Cabernet Sauvignon	Red Wine, Syrah or Shiraz
Anthocyanins	27.78 (23.20~76.51)	19.27 (0.06~74.47)	55.09 (55.09)	35.59 (12.08~51.12)	152.98 (152.98)
Malvidin	15.62 (1.24~54.14)	13.84 (0.00~53.57)	44.09 (44.09)	26.24 (8.67~37.97)	121.65 (121.65)
Peonidin	1.81 (0.25~8.09)	1.25 (0.02~5.03)	2.40 (2.40)	1.85 (0.70~2.66)	7.82 (7.82)
Petunidin	2.36 (0.34~6.18)	1.98 (0.02~5.66)	4.70 (4.70)	3.32 (1.21~4.78)	14.16 (14.16)
Dihydroflavonols	5.44 (4.58~5.98)				
Dihydromyricetin	4.47 (4.47)				
Flavanols	47.02 (11.35~113.11)	11.08 (0~56.31)	15.41 (15.41)	18.36 (18.18~19.48)	16.79 (16.79)
(+)-Catechin	6.81 (1.38~39.00)	7.14 (0.00~39.00)	6.21 (6.21)	7.70 (6.90~8.18)	6.82 (6.82)
(−)-Epicatechin	3.78 (0.00~16.50)	3.79 (0.00~16.50)	9.20 (9.20)	10.66 (10.28~11.30)	9.97 (9.97)
Procyanidin	35.41 (9.86~55.87)				
Flavanones	0.85 (0.78~0.94)	2.40 (1.30~3.50)			
Naringenin	0.05 (0.04~0.07)	1.77 (1.03~2.51)			
Flavonols	6.86 (2.02~15.40)	1.57 (0~6.68)	0.77 (0.20~1.07)	0.89 (0.05~1.74)	2.11 (2.11)
Quercetin	3.10 (0.79~7.31)	1.04 (0.00~3.36)	0.62 (0.14~0.84)	0.58 (0.02~1.21)	2.11 (2.11)
Flavones		0.17 (0~0.56)	0.06 (0.01~0.13)	0.04 (0.01~0.11)	
Total	87.95	34.53	71.33	54.88	171.88

**Table 2:** Clinical studies of flavonoids in allergic rhinitis.

Test Product	Major Flavonoid(S)	Study Design	Primary Endpoint	Ref.
Extract of *Perilla frutescents*	Rosmarinic acid (50 mg/day or 200 mg/day)	A 21-day randomized, double-blind, placebo-controlled study (n = 29)	A significant increase in responder rates for total symptoms related to seasonal allergic rhinoconjunctivitis	[66]
Apple polyphenols (500 mg/day)	Procyanidins, tannin, catechin, epicatechin, phlorizin, and chlorogenic acid	A 12-week randomized, double-blind, placebo-controlled study (n = 36)	A significant reduction in the sneezing score related to Japanese cedar pollinosis	[67]
Apple polyphenols (50 mg/day or 200 mg/day)	Procyanidins, phenol carboxylic acids	A 4-week randomized, double-blind, placebo-controlled study (n =33)	Significant improvements in sneezing attacks and nasal discharge in the 200 mg group and in sneezing attacks in the 50 mg group, related to persistent allergic rhinitis	[68]
Hop water extract (100 mg/day)	Quercetin, kaempferol glycosides	A 12-week randomized, double-blind, placebo-controlled study (n =39)	A significant difference in the symptom score and the symptom plus medication score related to Japanese cedar pollinosis 10 weeks after the intervention	[69]
Tomato extract (360 mg/day)	Naringenin chalcone	An 8-week randomized, double-blind, placebo-controlled study (n =33)	A significant decrease in the total symptom score related to perennial allergic rhinitis	[70]
EMIQ (100 mg/day)	Quercetin glycoside	An 8-week randomized, double-blind, placebo-controlled study (n = 20) (therapeutic design)	A significant decrease in the ocular symptom score related to Japanese cedar pollinosis	[71]
EMIQ (100 mg/day)	Quercetin glycoside	An 8-week randomized, double-blind, placebo-controlled study (n = 24) (preventive design)	A significant decrease in the ocular symptom plus medication score related to Japanese cedar pollinosis	[72]
Silymarin (420 mg/day)	Silibinin, silydianine, and silychristine	A 1-month randomized, double-blind, placebo-controlled study (n = 60)	A significant improvement in the clinical symptom severity related to allergic rhinitis	[73]
Pycnogenol (100 mg/day)	Proanthocyanidine	A 5 to 8-week randomized, double-blind, placebo-controlled study (n = 39) (preventive design)	Lower scores for the eye (−35%) and nasal (−20.5%) symptoms related to birch pollinosis	[74]

EMIQ, enzymatically modified isoquercitrin.

**Table 3:** Summary of the anti-allergic effects of flavonoids.

1. Biological properties
Antioxidant [9,10,13,24,25], anti-inflammatory [9,10,13,24,31,48], anti-allergic [28,29,30,31,32,33,34,35,36,37,38,39,40], and immune-modulating activities [31,40,42]
2. In vivo effects in animal models
Preventative and therapeutic beneficial effects of various flavonoids in several allergic models [53,54,55,56,57,58,59,60]
3. Epidemiological study
An increase of flavonoid intake is suggested to be beneficial for respiratory function [61,62,64,65]
4. Intervention study
Some kinds of flavonoids are efficacious for allergic rhinitis [66,67,68,69,70,71,72,73,74]
Pycnogenol is efficacious for asthma [75,76,77]
